# Photoinduced oxygen release and persistent photoconductivity in ZnO nanowires

**DOI:** 10.1186/1556-276X-6-404

**Published:** 2011-05-31

**Authors:** Jiming Bao, Ilan Shalish, Zhihua Su, Ron Gurwitz, Federico Capasso, Xiaowei Wang, Zhifeng Ren

**Affiliations:** 1 Department of Electrical and Computer Engineering, University of Houston, Houston, TX 77204, USA; 2 Department of Electrical and Computer Engineering, Ben Gurion University, Beer Sheva, Israel; 3 School of Engineering and Applied Sciences, Harvard University, Cambridge, MA 02138, USA; 4 Department of Physics, Boston College, Chestnut Hill, MA 02467, USA

## Abstract

Photoconductivity is studied in individual ZnO nanowires. Under ultraviolet (UV) illumination, the induced photocurrents are observed to persist both in air and in vacuum. Their dependence on UV intensity in air is explained by means of photoinduced surface depletion depth decrease caused by oxygen desorption induced by photogenerated holes. The observed photoresponse is much greater in vacuum and proceeds beyond the air photoresponse at a much slower rate of increase. After reaching a maximum, it typically persists indefinitely, as long as good vacuum is maintained. Once vacuum is broken and air is let in, the photocurrent quickly decays down to the typical air-photoresponse values. The extra photoconductivity in vacuum is explained by desorption of adsorbed surface oxygen which is readily pumped out, followed by a further slower desorption of lattice oxygen, resulting in a Zn-rich surface of increased conductivity. The adsorption-desorption balance is fully recovered after the ZnO surface is exposed to air, which suggests that under UV illumination, the ZnO surface is actively "breathing" oxygen, a process that is further enhanced in nanowires by their high surface to volume ratio.

## Background

Semiconductor nanowires provide a natural, ready-made structure in applications where small dimensions are required [1-3]. Their small diameter (≤100 nm) implies that a host of surface effects can influence their electrical and optical properties, which is important for the functionality and performance of nanowire-based devices [4-6]. Some observations such as enhanced gas sensitivity and photoconductivity, nowadays reconfirmed in ZnO nanowires [7-11], have been known in ZnO whiskers and thin films, and several attempts have been made to explain their occurrence [12-14]. The element, proposed here to tie together the various pieces of the puzzle into a single comprehensive model, is a fully reversible carbon-catalyzed photolysis, where carbon is omnipresent due to the pervasiveness of surface hydrocarbons, capable of exposing zinc on ZnO surfaces upon ultraviolet (UV) exposure. This surface effect is more pronounced and easily observed in structures of high surface-to-volume ratio, such as nanowires.

ZnO is a wide bandgap semiconductor material that has been attracting considerable research interest for many years. It has recently seen a renaissance due to reports of successful p-type doping [15], room-temperature ferromagnetism which could make it attractive for spintronic devices [16], and the large exciton binding energy which makes it attractive for photonics [17]. ZnO nanowire applications such as lasers, light-emitting diodes, nanogenerators, and field emitters have been reported [18-21]. In most of these applications, the typical surface sensitivity of the nanowire structure is often a disadvantage. However, ZnO nanowires are also finding use as gas sensors and UV detectors [7-11,22]. These nanowire sensors make use of the known surface sensitivity of ZnO which is further enhanced by the nanowire structure. The nature of this sensitivity has been controversial for over half a century [12-14,23,24]. Nanowires provide a new opportunity to look at the underlying mechanism of this surface sensitivity, which is the purpose of this work.

It has been known for many years that when ZnO films are exposed to above-bandgap (UV) illumination, their conductivity increases rapidly but persists long after the UV light is turned off [13]. This persistence has been shown to depend on the availability of ambient oxygen and has led to the suggestion of surface electron depletion region tightly related to the surface density of negatively charged adsorbed oxygen species () [13]. UV light causes this loosely bound oxygen to desorb from the surface at an increased rate, shifting the balance from adsorption to desorption. This reduces the surface electron depletion region leading to enhanced photoconductivity.

In vacuum, desorbed oxygen is pumped away. Therefore, this state of oxygen depletion may persist for as long as good vacuum is maintained. The density of these loose species of surface oxygen may be totally eliminated in vacuum, and therefore, one may expect under the same UV photon flux a somewhat higher conductivity to be reached in vacuum compared with that in air. The common observation is, however, of a much larger increase [7-11]. As we here report, the rate of photocurrent increase in ZnO nanowires associated with oxygen desorption in vacuum shows in fact two processes: one fast at a rate comparable to that in air and another one much slower that continues long after the first process has ended.

In the pioneering study on ZnO whiskers by Collins and Thomas [12], it was argued that the disappearance of the depletion region in vacuum alone cannot account for the large increase in conductivity and that a Zn-rich conductive layer, created by surface photolysis of lattice oxygen, was responsible for the large increase in photoconductivity observed in vacuum [12]. The large surface-to-volume ratio in the nanowire structure should enhance this effect, and indeed, several recent studies on ZnO nanowires have pointed out such increased photocurrent in vacuum [7-11].

In this work, we investigated photoconductivity of ZnO single nanowires in air and vacuum, and we found that the photoconductivity is much larger in vacuum than in air. We argued that this much enhanced photoconductivity arises from the decomposition of ZnO - surface photolysis of ZnO, and it cannot be explained by simple desorption of absorbed oxygen species. We further proposed that the ZnO photolysis is photocatalyzed by surface carbon, giving rise to the release of oxygen species in the form of carbon dioxide.

## Methods

ZnO nanowires in this study were synthesized by chemical vapor deposition using the vapor-liquid-solid technique [25]. Carbothermally reduced ZnO was used as the source material, and the gold nanoparticles were used as catalyst to seed and control the growth on a silicon substrate. Crystalline quality was assessed using transmission electron microscopy (TEM). ZnO nanowire photoconductors were prepared on an oxidized Si wafer with a 1-μm-thick silicon dioxide insulating layer. Electrical contacts were defined by electron-beam lithography and lift-off. They consisted of 5-nm thick of titanium and 50-nm thick of gold deposited sequentially using thermal evaporation. To achieve ohmic characteristics, the devices were then thermally treated for 10 min at 400°C in a mixed gas of 5% hydrogen and 95% helium at a total flow rate of 200 sccm.

Photoluminescence was excited using the 325-nm line of a He-Cd laser. Photoconductivity measurements were carried out at room temperature in a quartz-window optical cryostat, which can be filled with air or pumped to a vacuum of less than 10^-5 ^Torr. A high-pressure mercury lamp was used as a UV light source, and a bandpass filter (313-nm center wavelength, 10-nm bandwidth) was used to obtain monochromatic UV light from the mercury lamp.

## Results and discussion

**Figure 1:**
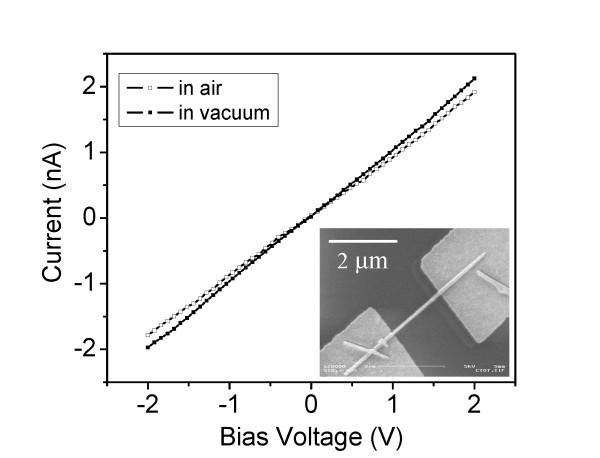
**Dark current versus voltage of the ZnO nanowire**. In air (unfilled squares) and in vacuum (filled squares). The measurement was performed after the device was kept in the dark for several days. Inset: SEM image of the device. The diameter of the wire is approximately 110 nm, and the gap between the electrodes in test is approximately1.8 μm.

A scanning electron microscopy (SEM) image of a typical ZnO nanowire device is shown in the inset of Figure 1. Seventeen of such devices were fabricated, and they showed similar performances. All data shown in this paper are from the same representative device. Under weak illumination (UV intensity <1 W/cm^2^), photoluminescence was found to be dominated by green emission, centered at approximately 2.15 eV. This luminescence is a ubiquitous feature of fine structured ZnO and has been recently suggested to originate at the ZnO surface [5]. Figure 1 shows the current-voltage (I-V) characteristics in the dark. The linear I-V relations indicate the desired Ohmic behavior of the contacts. The observed dark currents were low both in air and in vacuum, with a slightly greater value in vacuum.

**Figure 2:**
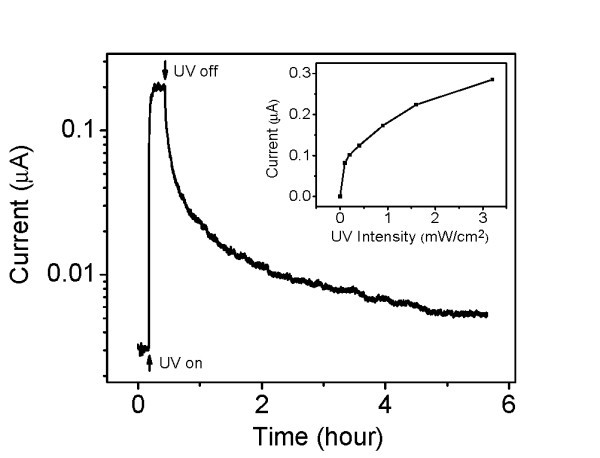
**Transient photocurrent of the ZnO nanowire in air under UV illumination**. The intensity of the λ = 313 nm light is approximately 1.3 mW/cm^2^. Inset: steady-state photocurrent versus light intensity in air. The bias voltage is 0.3 V and is the same for all other photocurrent measurements.

Figure 2 shows the time response of the photocurrent in air. Upon exposure to UV light, the photocurrent rises rapidly, reaching a steady-state value in several minutes. However, when the UV light is turned off, the current decays slowly following a short rapid decay. The overall decay is not exponential and slows down further over time. The current takes more than 10 h to return to the original dark value. The inset in Figure 2 shows the steady-state photocurrent as a function of light intensity. The current is clearly not a linear function of the intensity.

**Figure 3:**
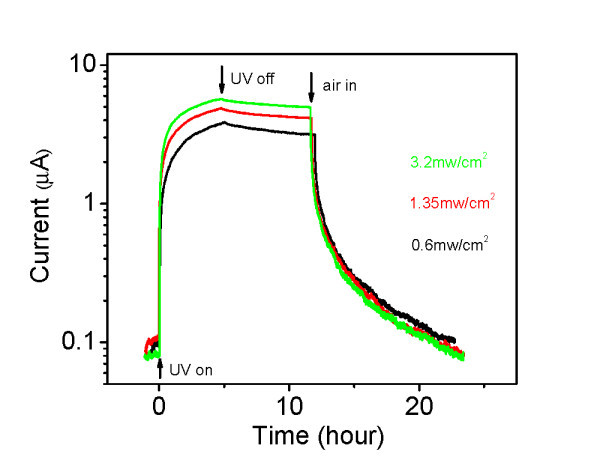
**Photoconductivity at three UV intensities in vacuum**. Same bias voltage and UV wavelength as in Figure 2. The steady-state currents have not been reached after about 5 h. The wire is kept in vacuum until air is let in after about 12 h (marked by a vertical arrow). The current at *t *= 0 is higher than that in Figures 1 and 2 because the UV was turned on before the dark current had reached its minimum.

A very different photoresponse is observed in vacuum. Figure 3 shows the photocurrent at three different UV intensities. Upon exposure to UV illumination, a short rapid photocurrent increase is observed for all the three intensities, followed by a slow increase. Steady state is not reached even after 5 h, although the photocurrents are already 20 times as large as those observed in air for the same intensity. When the light is turned off, the current shows only a small decay.

**Figure 4:**
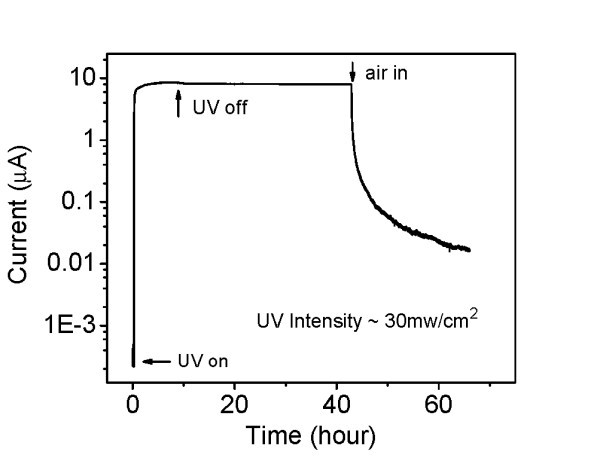
**Photoconductivity of the ZnO nanowire in vacuum when illuminated with multi-line UV light**. Light intensity is approximately 30 mW/cm^2^. The bias voltage is 0.3 V.

To obtain the maximum steady-state photocurrent in vacuum, we used the entire spectrum of the mercury lamp by removing the 313-nm bandpass filter. The total UV intensity above the ZnO bandgap was about 30 mW/cm^2^. The corresponding time response of the photocurrent is shown in Figure 4. A steady-state current of about 8.5 μA is reached after approximately 8 h of illumination. This current is not much larger than the currents in Figure 3, although the incident light intensity has been increased by an order of magnitude. As in Figure 3, the current follows a very slow decay pattern in vacuum, after the light is turned off, falling about 5% in the first day.

Persistent photoconductivity in ZnO has been observed in most of its known structures: thin films, microneedles, and nanowires (except, of course, for quantum dots because at least one dimension is required for conduction) [10,12,14,22]. Besides, by release of trapped electrons, carriers are also created through photogeneration of electron-hole pairs. To obtain an upper limit for this contribution, we note that the ZnO absorption length for light at 313 nm is comparable to the wire diameter (approximately 100 nm) and assume that all of the incident light is absorbed. Since ZnO is a direct bandgap semiconductor, the lifetime of photogenerated electron-hole pairs is shorter than 1 ns [26,27]. Taking for the lifetime 1 ns and assuming for the UV intensity the experimental value of ≈3.0 mW/cm^2^, the generated electron density is approximately 5 × 10^11^/cm^3^, and the corresponding photocurrent at 0.3 V bias voltage across the approximately 1.8 μm distance between two electrodes (see Figure 1) is about 2.5 × 10^-4 ^nA, assuming an electron mobility of approximately 20 cm^2^/Vs [9,10]. This current is about six orders of magnitude less than the dark current we observed. Optically excited carriers may decay into excitons which have a longer lifetime, but excitons do not contribute to the photoconductivity due to the charge neutrality. We therefore may safely neglect the contribution of photogenerated free carriers to the photocurrent in our case of weak illumination.

Undoped ZnO typically shows n-type conductivity, often suggested to be related to oxygen vacancies [7,9,10,12,28]. However, first-principles calculations showed that oxygen vacancies are not a shallow donor but rather a deep level [29]. Oxygen vacancies are more likely to be found close to surfaces, especially in the case of nanowires, and thereby to serve as surface traps [9,10,28,30,31].

Electron trapping associated with oxygen adsorption may be described by:(1)

where O_2_(g) and  indicate oxygen in its free and adsorbed states, respectively. The reverse process, desorption of oxygen from the surface, requires a photogenerated hole:(2)

Trapping of electrons charges the surface negatively, creating a non-conducting depletion layer under the surface. As previously discussed, a decrease or disappearance of this depletion layer under UV illumination underlies the photoconductivity of Zn nanowires. In the dark, reducing the oxygen pressure has only a minor effect on the adsorbed oxygen. This is evident in the rather minor change of conduction in vacuum from that in air prior to UV exposure, as shown in Figure 1.

**Figure 5:**
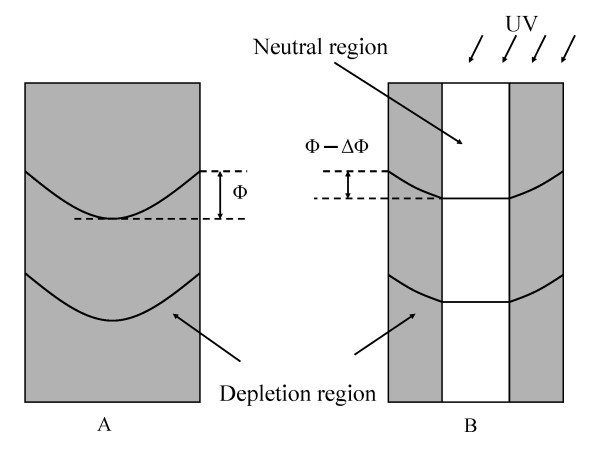
**Schematic of the depletion region in the dark (A) and under UV illumination (B)**. Photogenerated holes accumulate at the nanowire surface, partly neutralizing negatively charged absorbed oxygen species, which reduces the surface potential, leading to a reduction of the depletion width and increased photocurrent.

Figure 5 schematically illustrates the depletion layer profile in a ZnO nanowire in the dark and under illumination. As we observe, a rather low dark conductivity, compared with the photoconductivity under UV illumination, we assume that the wire is almost entirely depleted in the dark (Figure 5A). Later on, we shall justify this assumption quantitatively. The observed green subbandgap luminescence, centered at approximately 2.15 eV, has been previously suggested to be the result of surface Fermi level pinning at approximately 1.15 eV below the conduction band which implies a band bending potential, *Φ *≈ 1.15 V [30,31]. Once UV light is turned on, oxygen molecules are desorbed, as photoexcited holes become available, thereby reducing the surface potential *Φ *and the corresponding depletion width until a steady state is reached. Photoconductivity reflects the formation of a non-depleted core at the center of the wire, where the electron density is given by the doping level *n*. The changes of surface potential and the corresponding depletion width are determined by the interplay between oxygen adsorption and the net desorption rate which is a function of UV light intensity [32]. However, because of the relatively high oxygen partial pressure in air, total elimination of the depletion region and of the corresponding band bending would require extremely high illumination intensity. The maximum achievable photoconductivity should correspond to the native electron density *n *[33]. This explains why the saturation value of the photocurrent increases sublinearly with illumination intensity (Figure 2) [32,33].

As the illumination is turned off, adsorbed oxygen molecules trap electrons, gradually bending the bands, raising the surface potential barrier and the internal field. This reduces electron trapping at the surface, thereby reducing oxygen adsorption, and promotes spatial separation of electrons and photogenerated holes, thereby increasing their recombination lifetime. This positive feedback cycle qualitatively explains the persistence and non-exponential decay of the photocurrent as shown in Figures 2, 3, and 4.

In air, the discharging of the oxygen-related surface states and the resulting desorption of oxygen, as well as the shrinking of the depletion layer, require about 2 to 3 min, under UV illumination intensity on the order of 1 mW/cm^2^, as indicated by the time the photocurrent takes to reach its steady-state value. In vacuum, we basically have the same process, and therefore, it should be expected to take a roughly similar duration to establish a steady state. Of course, in vacuum, the steady state should be different, as there is no equilibrium between desorbed and adsorbed oxygen. Oxygen desorbs in vacuum at the same rate as in air, but since the oxygen is readily pumped out, it cannot be re-adsorbed [32]. Thus, the surface oxygen can be totally desorbed, totally eliminating the contribution of the adsorbed oxygen to the depletion and band bending. Indeed, in vacuum, we observe an initial rapid rise in the photoconductivity that reaches somewhat beyond the value reached in air (it rises to about 0.5 μA in the first 2 to 3 min). However, this transient is followed by a slower rise that continues for few hours and cannot be accounted for by the rapid process, in which loosely bound oxygen is desorbed. What could then explain this additional slow rise in vacuum?

To account for the slow response, we first note that this vacuum photoconductivity is not observed to saturate, even after hours of exposure, while in air, saturation is reached relatively fast, and that both air and vacuum photoconductivities are fully reversed upon exposure to oxygen in the dark. This suggests that the second, slower photoconductivity increase that we observe in vacuum is related to oxygen desorption as well. However, the slow and prolonged nature of this second process suggests that this oxygen is more tightly bound. Could it be lattice oxygen?

The idea of lattice oxygen desorption in ZnO was put forth to explain photoconductivity in whiskers [12]. It was suggested that the photoexcited holes, responsible for desorption of what we denote as loosely bound oxygen, are also responsible for subsequent lattice decomposition. This idea has not received enough attention since it is difficult to imagine that excess holes alone would be enough to decompose a lattice, held together by the high cohesive energy typical of oxide crystals. Nonetheless to date, several other stable materials, e.g., CdS, have been reported to show a so-called surface photolysis, where the anion was observed to be released upon exposure to above-bandgap illumination [34]. The question is therefore: why would such a process occur in ZnO?

Shapira et al. used mass spectrometry and Auger electron spectroscopy to identify the species desorbed from ZnO upon exposure to UV illumination in vacuum, as in our experiment [23]. They found that oxygen is desorbed from the surface of ZnO in the form of CO_2 _and suggested that surface hydrocarbons, commonly present on many solid surfaces, work in conjunction with the incident photon energy to release oxygen from the ZnO lattice, in a process that may be reversed by exposure to gaseous oxygen in the dark. Today, carbon is known to reduce oxides in what has been dubbed "carbothermal reduction" [35]. Carbothermal reduction is commonly used to enable decomposition of oxides at temperatures lower than their typical decomposition temperature, e.g., in ZnO nanowire growth [25]. It is therefore possible that if one changes the energy source from thermal to optical, carbon may still enhance oxide decomposition. In other words, we propose that when carbon is present on the surface, a "carbo-optical" reaction may be responsible for the slow oxygen desorption process we observe under UV exposure in vacuum.

We note, however, that although hydrocarbons are almost always present, it is not absolutely clear whether the presence of carbon is critical for ZnO photolysis, as there has also been a single report of O_2 _photodesorption from ZnO [24]. We also note that it may be possible that oxygen containing compounds other than O_2_, e.g., H_2_O, could serve as oxygen source to replenish the lost oxygen [36], although CO_2 _was clearly found ineffective [23]. Nonetheless, it is clear that only oxygen can take the place of the lost lattice oxygen and restore the resistivity, regardless of the actual chemical species supplying it. Finally, it was proposed that two electron-hole (excitons) pairs could provide enough energy to photodecompose lattice ZnO [28]. However, firstly, this model is partial as it does not require carbon, and if it actually worked, we should be able to detect emission of oxygen, while in fact it is CO_2 _that is actually detected. Secondly, a process requiring two electron-hole pairs to be perfectly timed to act together as one is of very low probability, if at all possible. On the other hand, the same process involving carbon, as we propose, should require less energy and would easily account for the observed CO_2 _emission.

Free electrons released from desorbed oxygen, as well as from the Zn-rich surface layer, should remain free as long as the ZnO nanowire is maintained in perfect vacuum, leading to indefinitely persistent photoconductivity. The minor decay of photocurrent we observe in vacuum clearly reflects the residual oxygen and is thus a rough indicator of our vacuum quality.

Finally, we shall now support our previous assumption of nearly total depletion. The maximum photocurrent in air that we were able to achieve was *I*_ph _= *S eμ *Δ*n E *≈ 0.5 μA, where *S *≈ 10^-10 ^cm^2 ^is the typical cross-sectional area of our nanowires, *e *is the electron charge, and *E *= 1,700 V/cm the electric field. Assuming a mobility,*μ *≈ 20 cm^2^/Vs, we get an excess electron density Δ*n *≈ 9.2 × 10^17^cm^-3^, which is the order of magnitude of the electron density of undoped ZnO nanowires without surface charge trapping [9,28]. The critical wire diameter *d*_crit_, below which a nanowire will be completely depleted by surface states, is [6]:

where *ε *is the permittivity of ZnO (*ε *is approximately 8.5) [12]. Assuming *ϕ *= 1.15 V, as previously suggested, we obtain *d*_crit _≈ 100 nm. This value is about the actual diameter of the ZnO nanowire. The nanowire is then likely to be near total depletion, in agreement with its low dark conductivity.

## Conclusions

In summary, we proposed a model to account for the observed persistent photoconductivity in ZnO nanowires, which ties together several previously suggested explanations of different facets of the problem into a single comprehensive picture. Negatively charged traps associated with adsorbed oxygen deplete ZnO nanowires of electrons. This oxygen-related depletion is partially undone by exposure to UV in air and completely reversed by UV exposure in vacuum. UV exposure in air removes loosely bound oxygen and in vacuum further removes lattice oxygen in a process that may be catalyzed by surface hydrocarbons. According to the suggested model, carbon-catalyzed photolysis is responsible for the slow release of lattice oxygen, exposing zinc on ZnO surfaces upon UV exposure in vacuum or low oxygen environment. This effect is more pronounced in structures of high surface-to-volume ratio like nanowires. This oxygen removal, however, is fully reversible upon exposure to oxygen in the dark, in a process that is somewhat reminiscent of breathing. We note that the role of loosely bound oxygen in inducing electron surface traps could possibly be assumed by oxygen containing molecules, e.g., water, which could also serve to reverse the slow photolysis taking place in vacuum.

## Competing interests

The authors declare that they have no competing interests.

## Authors' contributions

JMB performed the photoconductivity measurements and prepared the draft. ZS performed photodesorption measurement. ZFR and XW grew ZnO nanowires. FC conceived the study, participated in its design and coordination, and helped to revise the manuscript. IS and RG proposed carbothermal photodecomposition of ZnO and helped to revise the manuscript. All authors read and approved the final manuscript.
